# Three discipline collaborative radiation therapy (3DCRT) special debate: The single most important factor in determining the future of SBRT is immune response

**DOI:** 10.1002/acm2.12728

**Published:** 2019-10-01

**Authors:** Clemens Grassberger, Kathryn Huber, Naduparambil K. Jacob, Michael D. Green, Peter Mahler, Joann Prisciandaro, Michael Dominello, Michael C. Joiner, Jay Burmeister

**Affiliations:** ^1^ Department of Radiation Oncology Massachusetts General Hospital Boston MA USA; ^2^ Department of Radiation Oncology Tufts Medical Center Boston MA USA; ^3^ Department of Radiation Oncology The Ohio State University Columbus OH USA; ^4^ Department of Radiation Oncology University of Michigan Ann Arbor MI USA; ^5^ Department of Human Oncology University of Wisconsin Madison WI USA; ^6^ Department of Oncology Wayne State University School of Medicine Detroit MI USA; ^7^ Gershenson Radiation Oncology Center Barbara Ann Karmanos Cancer Institute Detroit MI USA

## 1 THREE DISCIPLINE COLLABORATIVE RADIATION THERAPY (3DCRT) DEBATE SERIES

Radiation Oncology is a highly multidisciplinary medical specialty, drawing significantly from three scientific disciplines — medicine, physics, and biology. As a result, discussion of controversies or changes in practice within radiation oncology involves input from all three disciplines. For this reason, significant effort has been expended recently to foster collaborative multidisciplinary research in radiation oncology, with substantial demonstrated benefit.^1^, ^2^ In light of these results, we endeavor here to adopt this “team‐science” approach to the traditional debates featured in this journal. This article is part of a series of special debates entitled “Three Discipline Collaborative Radiation Therapy (3DCRT)” in which each debate team will include a radiation oncologist, medical physicist, and radiobiologist. We hope that this format will not only be engaging for the readership but will also foster further collaboration in the science and clinical practice of radiation oncology.

## 2 INTRODUCTION

While stereotactic body radiotherapy (SBRT) has demonstrated considerable success in providing local tumor control, regional and systemic progression remains a problem. Overcoming this problem by harnessing the immune response is an intriguing possibility and there is hope for synergism when combining SBRT and immunotherapy that may result in a clinical benefit for some patients. However, immunotherapy when combined with radiotherapy has thus far met with mixed success. Will a greater understanding of the immune response and abscopal effect allow us to unlock an even greater potential future for SBRT? The topic of this month’s 3DCRT debate is whether immune response is the most important factor determining the future of SBRT.

Arguing for the proposition will be Drs.Clemens Grassberger, Kathryn Huber, and Naduparambil Jacob.

Clemens Grassberger, PhD, is an Assistant Professor at Harvard Medical School and Massachusetts General Hospital, and head of the laboratory for radiation‐drug treatment design. He studies the effects of radiation in combination with targeted therapies, and how radiotherapy can be used to delay resistance development or modulate the patient’s immune response.

Kathryn Huber, MD, PhD, is a Radiation Oncologist at Tufts Medical Center and Assistant Professor at Tufts University School of Medicine. She specializes in the treatment of thoracic, breast, and head and neck cancers and is the Director of Radiobiology for the residency training program at Tufts.

Naduparambil Jacob, PhD, is an Associate Professor in the Department of Radiation Oncology at The Ohio State University Comprehensive Cancer Center. His laboratory focuses on developing biomarkers for minimally invasive radiation biodosimetry, early detection and mitigation of delayed effects. He also seeks to develop strategies for better radiosensitization of cancer cells, protecting normal tissues to achieve better therapeutic ratio.

Arguing against the proposition will be Drs. Michael Green, Peter Mahler, and Joann Prisciandaro.

Michael Green, MD, PhD, is an Assistant Professor at the University of Michigan in the Department of Radiation Oncology. His group utilizes expertise in quantitative immunophenotyping, oxidized lipidomics, radiobiology, and metabolism to define and harness the determinants of inflammation which shape anti‐tumoral immunity and influence radiotherapy and immunotherapy efficacy.

Peter A. Mahler MD,PhD, is a Clinical Professor of Human Oncology at the University of Wisconsin School of Medicine and Public Health, Madison, Wisconsin. He has a particular interest in palliative medicine and radiotherapy, and in normal tissue response to radiation.

Joann Prisciandaro, PhD, is a Clinical Professor at the University of Michigan in the Department of Radiation Oncology. She has a strong interest in brachytherapy, education, and radiation safety.

## 3 OPENING STATEMENTS

### 3.1 Clemens Grassberger, PhD; Kathryn Huber, MD, PhD; Naduparambil Jacob, PhD

Stereotactic body radiotherapy (SBRT) has proven to be one of the most significant recent advances in the delivery of radiation. Its accuracy in tumor targeting allows the sparing of normal tissues such that ablative doses can be often administered with few side effects. These characteristics have made it an integral part of definitive approaches in lung, liver, prostate, and pancreatic cancer. The treatment of nonsmall cell lung cancer (NSCLC) has particularly been transformed by the use of SBRT in early as well as late stage disease, and we will use this large population as a case study for how, in our opinion, immune response will shape the future of SBRT:

#### 3.1.1 Early‐Stage NSCLC

Early‐stage patients with NSCLC have an unacceptably high rate of regional nodal or distant recurrence, on the order of 20–25% at 3 yr, even though the vast majority of patients are locally controlled.^3^ Yet chemotherapy has been shown not to be beneficial for stage I NSCLC patients with tumors under 4 cm.^4^ In contrast, immunotherapy has demonstrated encouraging clinical results, superior to standard cytotoxic chemotherapy, for patients with locally advanced or metastatic disease,^5^, ^6^, ^7^ and is now also being studied in the adjuvant setting after SBRT for early‐stage NSCLC.^8^, ^9^ Compelling evidence has shown that radiation can enhance the antitumor immune response; however, this happens inconsistently.^10^ The high doses per fraction delivered in SBRT regimens may help generate robust immune responses, and although the dose effect for inducing DNA breaks is deterministic in nature, recent preclinical studies indicate the existence of a dose per fraction threshold for best immune responses.^11^, ^12^

It appears that we are able to achieve very high local control with a variety of fractionation regimens,^13^ but further exploration into which of these elicit the most robust synergy with immunotherapy could prove to be the main determining factor of long‐term survival. Since the acute inflammatory response can be causative of delayed and late toxicities, resulting immune response need not be all that beneficial to achieve better therapeutic ratio and better quality of life in surviving patients.

#### 3.1.2 Metastatic NSCLC

In addition to the standard role of SBRT in the treatment of early‐stage, medically inoperable NSCLC, there is an emerging evidence that SBRT can improve survival in patients with oligometastatic disease.^14^, ^15^ Two randomized studies investigating the role of stereotactic radiation for the comprehensive treatment of oligometastatic cancers were presented at the 2018 ASTRO Annual meeting, providing the strongest evidence to date for the use of SBRT for oligometastatic cancer.^16^, ^17^ For NSCLC, the authors report an impressive increase in Progression Free Survival (14.2 vs 4.4 months) with the addition of SBRT to standard management, with an associated Overall Survival advantage of 41 vs 17 months.^16^

However, even with these impressive results, the majority of patients have eventual distant progression that dictates the course of their disease. The combination of immunotherapy and fine‐tuned radiation to the optimal volume, dose, and fractionation that amplifies the antitumor immune response has the potential to dramatically expand the population who benefit from SBRT.

#### 3.1.3 From cell kill to immune response

SBRT has already been shown to spare the circulating lymphocytes compared to conventional fractionation in patients.^18^ Especially in the context of combining radiation with immunotherapeutic approaches, radiation‐induced depletion of lymphocytes can dampen synergistic effects,^19^ and it has been shown that the predictive value of lymphocyte counts also holds for metastatic patients on treatment with checkpoint inhibitors receiving RT.^20^

In the metastatic setting, when SBRT is combined with immunotherapeutic approaches, the focus on cell kill and other biological factors is certainly reduced, while inducing a robust and lasting immune response is paramount. This will present a paradigm shift away from SBRT regimens that maximize cell kill toward approaches that focus on immune response and preservation, with the potential to change the role of SBRT in clinical practice in terms of sequencing, prescribed dose, and fractionation.

#### 3.1.4 Other factors relevant to SBRT

The last two decades have shown technical advances which preceded and enabled the clinical implementation of SBRT: online imaging, accurate dose calculation, and the resulting reduction of margins were crucial for the development of SBRT. With the advent of MRI‐LINAC systems, which enable imaging even during treatment, we feel that the technical factors enabling more accurate delivery have reached a plateau, and that the factors determining the future of SBRT are biological.

Aside from the immune response there are certainly other biological phenomena which might be important in the therapeutic efficacy of SBRT, among them are hypoxia and vascular effects. The data to support the indirect cell killing attributed to vascular damage following SBRT are beyond the scope of this debate, and we refer the reader to the following review articles.^21^, ^22^ However, it appears that the impact of these secondary cytotoxic mechanisms are largely local and any impact beyond local sensitization circles back to stimulation of the anticancer immune response by allowing the release of tumor‐associated antigens, DNA fragments, and proinflammatory cytokines into the circulation.

Therefore, given the emerging benefit of combining SBRT and immunotherapy outlined in our argument above, and the developing work demonstrating that SBRT can impact systemic disease control, even in early stages of disease, we are convinced that “the single most important factor determining the future of SBRT is immune response”.

### 3.2 Michael Green, MD, PhD; Peter Mahler, MD, PhD; Joann Prisciandaro, PhD

Stereotactic Body Radiation Therapy (SBRT), also known as Stereotactic Ablative Radiotherapy (SABR), is characterized by the precise delivery of high doses of focal radiation typically to extracranial lesions over the course of five or fewer treatment fractions.^23^ Although the earliest publications on SBRT emerged in the mid to late 1990s,^24^, ^25^, ^26^, ^27^, ^28^ it was not until the new millennium that this treatment approach began to take root in the radiation oncology community.

SBRT evolved from the success of brain stereotactic radiosurgery (SRS), a treatment technique that stereotactically delivers high doses of radiation to precisely defined cranial lesions.^29^ The extension of stereotactic treatments extracranially required tools to ensure patients were appropriately immobilized, the target position was verified directly or through the use of surrogates (e.g., fiducial markers, neighboring anatomy), and target motion was minimized, as well as a delivery system that was capable of precisely delivering radiation to the intended target. This was made possible through advances in target localization with the availability of high‐resolution computed tomography (CT) images, respiratory correlated CT images (4DCT, or slow CT scanning in the early 2000s), magnetic resonance imaging (MRI), and functional imaging (e.g., functional MRI, Positron Emission Tomography/CT (PET/CT), and PET/MRI).^30^ Advancements were also made possible through the development of motion management tools (e.g., abdominal compression, breath hold, gating, and tracking techniques), improved immobilization systems (a summary of in‐house and commercial systems may be found in Benedict et al.,^31^) in‐room monitoring (e.g., cone beam CT), and real‐time adaptive planning with systems such MR‐guided radiotherapy units (e.g., the ViewRay Tri‐60‐Co/MRI and MRIdian units (ViewRay Inc., Oakwood Village, OH)).

Further technological advances in SBRT are being realized through the introduction of volumetric‐modulated arc therapy (VMAT) and flattening filter‐free (FFF) beams which allow for rapid treatment delivery compared to conventional radiotherapy. A reduction in delivery time may assist in minimizing intrafraction motion, resulting in an improvement in target reproducibility, and a reduction in the uncertainties associated with target delineation. Given the high dose per fraction delivered with SBRT treatment, a reduction in target to block the margin is essential to minimize the risk of complications and toxicity to neighboring critical structures.^25^

With these advances, increases in dose per fraction with a concomitant decrease in fraction number has become possible. Stereotactic body radiotherapy is now becoming the standard of radiation treatment for metastatic brain tumors,^32^ and has demonstrated an increase in progression‐free survival (PFS) for nonoperable, early stage lung cancer patients.^33^ SBRT is also seeing increased use in treating prostate cancer, with early reports of increased efficacy and decreased toxicity.^34^ Trials are underway to evaluate SBRTs efficacy in other disease sites, including advanced lung cancer, pancreatic cancer, sarcoma, and oligometastatic disease. Stereotactic body radiotherapy is also an attractive option in a re‐irradiation setting for spinal, thoracic, and head and neck cancers.^35^, ^36^, ^37^, ^38^, ^39^

The clinical utilization of immunotherapy has been a relatively recent development, and significant research efforts are underway to define primary and acquired resistance mechanisms. Immunotherapy is fundamentally altering treatment paradigms in almost all disease histology. Immune checkpoint blockade, the most clinically utilized form of immunotherapy, is capable of producing durable treatment responses, but only a minority of patients benefit. There is considerable excitement and hope that we can wed the immune modulatory effects of SBRT and immunotherapy to benefit even more patients. But it is myopic to believe that the only future of SBRT is due to its hoped‐for immune modulatory effects.

Many avenues of immunotherapy are being pursued. The most frequently used drugs involve checkpoint inhibitors such as PD‐1 drugs: [Pembrolizumab (Keytruda), Nivolumab (Opdivo), Cemiplimab (Libtayo)], and PD‐L1 inhibitors [Atezolizumab (Tecentriq), Avelumab (Bavencio), and Durvalumab (Imfinzi)]. Checkpoint inhibitors targeting CTLA‐4 such as Ipilimumab (Yervoy) and Tremelimumab are also clinically utilized, but these agents cause an increased frequency and grade adverse effects as compared to anti‐PD‐1 axis agents.

In a recent landmark immunotherapy study, Wolchok and colleagues found that in metastatic melanoma, combination therapy of Ipilimumab and Nivolumab improved the 3‐year overall survival to 58% from 32% with Ipilimumab alone.^40^ Importantly, combination therapy, even in one of the most immunogenic tumor histologies, failed to demonstrate even an additive benefit while substantially increasing severe side effects. Immune evasion is not the only hallmark of cancer, and immunotherapy will not be a panacea for all patients, but rather an important tool for the subset of patients who have strong, pre‐existing antitumoral immunity.^41^, ^42^ Of note, immunotherapy administration may be accompanied by severe, even life‐threatening toxicities. This makes them unlikely candidates for treatment of tumors such as prostate cancer, where good, albeit not yet perfect, therapies exist.

SBRT can indeed invoke significant inflammatory responses, eliminate immunosuppressive elements, and augment T‐cell immunity in preclinical models.^43^ However, clinically, abscopal responses with current generation immunotherapy are observed at approximately the same frequency in human patients as Sasquatch is sighted. In prostate cancer, randomized trials have shown SBRT to improve the overall survival of metastatic patients, and they have also demonstrated a lack of benefit of SBRT to potentiate ipilimumab efficacy.^44^, ^45^ Stereotactic body radiotherapy has incredible power and utility outside immune modulation. It causes vascular collapse, causes cell cycle arrest, and can promote tumor cell death.^46^ It will continue to serve as an attractive treatment option for cancer patients regardless of their immunologic status or response to immunotherapy.

SBRT is an attractive, focal treatment option. It is noninvasive, and given its dose and fractionation schedule, has been shown to be more potent and convenient than conventional radiotherapy.^47^ Randomized trials have now shown that SBRT can improve the overall survival of oligometastatic patients, demonstrating that it has utility in both localized and disseminated disease settings.^17^ However, identifying the single most influential factor to the future of SBRT is daunting. The future success of this technique will most likely be linked to advances in early detection and understanding of disease progression, target delineation, radiobiological modeling, and dose accumulation within treatment planning systems, precision delivery, adaptive therapy, machine learning, radiomics, and systemic therapies, potentially including immunotherapy. While a synergetic effect between SBRT and immunotherapy may exist, thus far this has been demonstrated only in a limited subset of patients.

## 4 REBUTTAL

### 4.1 Clemens Grassberger, PhD;Kathryn Huber, MD, PhD; Naduparambil Jacob, PhD

We agree with our fellow discussants on multiple points. They give an excellent overview of the scientific and technical advances that paved the way for the clinical adaptation of SBRT and are the foundation of its status today as an important part of cancer treatment. We also concede that prostate cancer might not be the site where the immune effects of SBRT will prove crucial, given the relatively low mutation load and immunogenicity, the high rates of diagnosis in early stage, and the excellent disease‐specific survival.^48^

In this sense, we are as guilty of bias as our fellow discussants: in our original argument we have used lung cancer to make our case, which is as suitable to our line of argumentation as prostate cancer is to the counterargument.

However, our conviction that the immune response is the most important factor for the future of SBRT does not stem from indication‐specific reasoning. We agree that the current, important position that SBRT occupies is built on the developments mentioned. However, for the future of SBRT we base our assessment on two indication‐independent observations:

1. Advances in immunotherapeutic approaches for metastatic cancers will lead to an increased use of SBRT.
2. Agents that provide significant benefit over traditional cytotoxic chemotherapy in metastatic cancer will be integrated into treatment of earlier stages of disease.

Regarding the first observation: Unless immunotherapy leads to response rates close to 100%, it will likely be complemented with other approaches, among them SBRT. Recent data by Formenti et al. show encouraging results in lung cancer patients treated with Ipilimumab and SBRT,^49^ where anti‐CTLA‐4 agents had failed to demonstrate significant efficacy alone^50^ or in combination with chemotherapy.^51^ As of 2018 the combination of RT (majority SBRT) with anti‐PD‐1/PD‐L1 agents alone was being tested in 114 trials^52^, demonstrating the impressive extent of activity in this field.

The results of one of the first of these trials coming to publication, PEMBRO‐RT, which is a phase 2 randomized control trial in patients with metastatic NSCLC, provides promising information on identifying patients who may benefit most from adding SBRT to immunotherapy. They found a doubling of the overall response rate, from 18% in the Pembrolizumab arm to 36% in the experimental arm of patients who were treated with SBRT to a single metastatic site in addition to Pembrolizumab. Although this did not meet the goal of the trial to achieve a 50% response rate, it corresponded with a doubling of the median overall survival from 7.6 months to 15.9 months. Most interestingly, the subgroup analyses showed the largest benefit from the addition of SBRT was seen in patients with PD‐L1–negative tumors, supporting the concept that radiation can activate previously nonimmunogenic NSCLC.^53^

In the second observation, we refer to situations where an immunotherapeutic approach combined with SBRT might be integrated into treatment of early stage or locally advanced disease. An interesting example is the combination of checkpoint inhibitors + SBRT for preoperative treatment of high‐risk breast cancer.^54^ With the complete removal of the tumor post‐SBRT, the sole purpose of this preoperative regimen is to modulate the immune response to prevent systemic recurrence following definitive local treatment. If trials like this show decreased distant failure, the utilization of SBRT would expand dramatically as an adjuvant therapy rather than its current status as only a local tumor treatment.

What unites us with our fellow discussants is the belief in the importance of SBRT to radiation oncology and that its future is bright. Which factors will determine this future is indeed an open question. However, we strongly disagree with the suggestion that the abscopal effect of radiation is mythological, like the Sasquatch. We rather see similarities to the “learning from exceptional responders” paradigm in drug development,^55^ where studying unusual responses has led to discovery of unknown mechanisms and enriched both patient care and science.^56^ We believe that through experimentation and clinical observations, we will come to greater understanding of the abscopal effect and unlock its full potential.

### 4.2 Michael Green, MD, PhD; Peter Mahler, MD; Joann Prisciandaro, PhD

We agree with our colleagues that the promise of SBRT and immunotherapy is quite exciting. The hope that a focal treatment can control local disease and amplify systemic therapy responses has long been a dream of radiation oncologists. Quoting a palliative care colleague: “It is okay to hope. Everyone deserves to hope. But everyone needs a plan, and hope is not a plan.” Data, not hope is required to guide the management of our patients, and our colleagues were able to point to limited data in one immune responsive histology, NSCLC, in an attempt to support their argument.

However, we remind our colleagues that SBRT offers excellent local control independent of disease histology and antitumoral immunologic status in early stage disease. We agree that SBRT is now an integral part of definitive care in lung, liver, prostate, and pancreatic cancer.^57^, ^58^ We also remind our colleagues that there are presently no indications for immunotherapy in prostate and pancreatic cancer, suggesting that SBRT acts independently of immune responses.^59^Tumoral ablation with SBRT is sufficient in the majority of early stage patients to cure them of disease.

We agree that SBRT can improve survival in patients with oligometastatic disease. Again, this finding has been demonstrated without combining SBRT with immunotherapy. We also agree that frequent distant progression represents a common form of failure in these patients. While we share the hope of our colleagues that immunotherapy could alter the frequency of this mode of failure, we are unaware of any quantitative patterns of failure following immunotherapy administration to support this claim. Stereotactic body radiotherapy can offer effective salvage options for the management of disease in immune privileged sites such as the brain in patients receiving immunosuppressive steroids, further highlighting the independence of SBRT and immune responses.

Our colleagues emphasize that SBRT has been shown to decrease loss of circulating lymphocyte populations compared to conventional radiation. We agree that normal tissue sparing is a benefit to SBRT. Such lymphocyte preservation may be a necessary condition for synergy, but is unlikely to be a sufficient one. Randomized trials of supplementation with granulocyte colony stimulating factor to prevent neutropenia have failed to show differences in the rates of febrile neutropenia or survival of patients.^60^ By analogy, it seems quite possible that lymphocyte preservation may not increase immunotherapy effectiveness.

We believe that one key to future advances in SBRT will be driven by improvements in the understanding of tumor biology. There are diverse tumor cell and microenvironmental factors which influence treatment trajectory and treatment response. Immunotherapy is one such facet that requires additional study.^42^ For example, much research is ongoing to investigate the “vaccine‐like” effect of radiation.^61^ Genomics continues to inform our understanding of the mutational and transcriptional drivers of cancer.^62^ Examination of immune‐independent mechanisms of radiotherapy efficacy including vascular normalization require further study.^63^

A second key to future progress in SBRT is continued advancements in imaging, targeting, and adaptive therapy. These will allow for further dose escalation which has the potential to enhance the radiation therapeutic ratio, as well as enable additional treatment sites to benefit from SBRT.^64^ Deeper examination of the modality of radiation used for SBRT delivery (e.g., photon versus proton) continues.^65^ Furthermore, the advent of ultra‐high dose FLASH radiotherapy suggest that a deeper examination of dose rate is important to improve SBRT efficacy.^66^ Finally, we have only begun to utilize machine learning and quantitative imaging to better delineate and target disease.^67^

We remain unconvinced that “the single most important factor determining the future of SBRT is immune response.” The majority of cancers are not immunogenic, and patients failing immunotherapy represent the single largest oncologic patient population at present. Implying that immune stimulation is the most important mechanism of SBRT is intriguing, but there is limited clinical data to support this hypothesis. Stereotactic body radiotherapy is an effective treatment modality regardless of immunotherapy, and this understanding should drive future indications for its utilization.

## CONFLICTS OF INTEREST

The authors declare no conflicts of interest.
